# Official Advice

**Published:** 1921-06-11

**Authors:** 


### OFFICIAL ADVICE.

We are glad to note that the Ministry of Health have at length deliberately stated their policy in connection with the great venereal question. On the whole, their policy is one of half-measures, though possibly, on such a bitterly controversial subject, any other would meet with determined criticism (see p. 181). Their circular clears up, however, such doubtful points as the continuation of experimental ablution centres, the legal position of chemists vending disinfectants against venereal disease so long as these substances are not sold accompanied by printed recommendations for their use. Also a clear statement of this type should form a necessary basis for co-operation between societies and persons to help the Government in a very urgent campaign.

The Interdepartmental Committee which considered the question wisely drew a large part of its evidence from war experience. Preventive treatment by a skilled assistant, after exposure to possible infection, gave invariably better results than when the same measures were taken by the individual himself, however carefully instructed. There was general agreement that the best way to avoid infection is to abstain from incurring the risk, and that disinfection after exposure is effective only when thoroughly applied. Considering the moral and social side of the problem also, the Ministry have decided that they cannot make a system of self-disinfection for the civil community part of the official policy.

On the other hand, they urge that local authorities should undertake to further general public instruction and enlightenment. In outline this teaching should point out that the best way to avoid infection is to avoid the risk of it. If this risk has been incurred, then it is the persons' duty to themselves and the community immediately to take cleansing precautions. If any signs or symptoms of the disease, however slight, are experienced the individual should promptly seek medical advice, for self-treatment is more than likely to be disastrous. It is impossible to say that in this statement we see a new departure and a great advance in dealing with the venereal disease question, but it gives a middle-course policy based upon which much valuable work can be done.

